# miR-100-3p inhibits cell proliferation and induces apoptosis in human gastric cancer through targeting to BMPR2

**DOI:** 10.1186/s12935-019-1060-2

**Published:** 2019-12-27

**Authors:** Chun-Wei Peng, Ling-Xiao Yue, Yuan-Qin Zhou, Sai Tang, Chen Kan, Lei-Ming Xia, Fan Yang, Si-Ying Wang

**Affiliations:** Department of Pathophysiology, School of Basic Medicine, Anhui Medical University, 81 MeiShan Road, Hefei, 230032 China

**Keywords:** Gastric cancer (GC), miR-100-3p, Bone morphogenetic proteins receptor (BMPR2), B cell lymphoma-2 (Bcl2)

## Abstract

**Background:**

miR-100 has been reported to closely associate with gastric cancer (GC) initiation and progression. However, the underlying mechanism of miR-100-3p in GC is still largely unclear. In this study, we intend to study how miR-100-3p regulates GC malignancy.

**Methods:**

The expression levels of miR-100-3p in vitro (GES-1 and GC cell lines) and in vivo (cancerous and normal gastric tissues) were examined by quantitative real-time PCR (qRT-PCR). MTT and PE/Annexin V analyses were responsible for measurement of the effects of miR-100-3p on GC cell proliferation and apoptosis. Transwell assay with or without matrigel was used to examine the capacity of migration and invasion in GC cells. The interaction of miR-100-3p with bone morphogenetic protein receptor 2 (BMPR2) was confirmed through transcriptomics analysis and luciferase reporter assay. qRT-PCR and Western blot analyses were applied to determine the expression of ERK/AKT and Bax/Bcl2/Caspase3, which were responsible for the dysfunction of miR-100-3p.

**Results:**

miR-100-3p was down-regulated in GC cell lines and cancerous tissues, and was negatively correlated with BMPR2. Loss of miR-100-3p promoted tumor growth and BMPR2 expression. Consistently, the effects of miR-100-3p inhibition on GC cells were partially neutralized by knockdown of BMPR2. Over-expression of miR-100-3p simultaneously inhibited tumor growth and down-regulated BMPR2 expression. Consistently, over-expression of BMPR2 partially neutralized the effects of miR-100-3p over-expression. Further study demonstrated that BMPR2 mediated the effects downstream of miR-100-3p, which might indirectly regulate ERK/AKT and Bax/Bcl2/Caspase3 signaling pathways.

**Conclusion:**

miR-100-3p acted as a tumor-suppressor miRNA that down-regulated BMPR2, which consequently inhibited the ERK/AKT signaling and activated Bax/Bcl2/Caspase3 signaling. This finding provided novel insights into GC and could contribute to identify a new diagnostic and therapeutic target.

## Background

Gastric cancer (GC) is still a clinically challenging cancer worldwide. More than 1,000,000 new cases have been diagnosed in 2018, with an estimated death toll of 783,000, making it the fifth most common cause of cancer and cancer deaths [1]. *Helicobacter pylori* (HP) infection is a major risk factor for GC, to which nearly 90% of non-cardia GC is attributed [2, 3]. In addition, increased intake of preserved foods, low intake of fruits, drinking and smoking are also identified risk factors [4, 5]. Due to the diagnosis and treatment of GC have been improved, the 5-year survival rates for stage IA and IB tumors treated with surgery are 94% and 88%, respectively. On the other hand, stage IIIC tumors treated with surgery has a 5-year survival rate of only 18% [6]. Thus, understanding the underlying mechanisms of GC is critical to GC screening and treatment.

MicroRNAs (miRNAs) are short (about 18–25 nucleotides) endogenous non-coding RNAs, which regulate gene expression at post-transcriptional level to promote mRNA degradation and repress translation, by binding to the 3′- untranslated region (UTR) of targets genes [7, 8]. Each miRNA precursor can be cleaved into two mature molecules, namely miR-5p and miR-3p, which have different recognition zones with different functions [9]. Lots of research indicated that miRNA were closely correlated with tumor cells apoptosis and proliferation [10, 11]. Targeting to the specific miRNAs sheds new light on anti-cancer treatments.

It has been shown that miR-100 was dysregulated in the GC, as a tumor suppressor or oncogene [12, 13], detailed mechanism underlying this dysfunction is still unknown. miR-100-5p has been reported to be down-regulated in GC [12], however, the function of miR-100-3p in GC is urgent to discover.

In this study, we found that miR-100-3p acted as a tumor-suppressor. Further, it could down-regulate BMPR2, which consequently inhibited the ERK/AKT signaling and activated Bax/Bcl2/Caspase3 signaling. This finding provided novel insights into GC and could contribute to identify a new diagnostic and therapeutic target.

## Materials and methods

### Tissue sample collection

GC tissues and paired normal gastric tissues were harvested from the First affiliated Hospital of Anhui Medical University from 2016 to 2017.Tissues were immediately frozen in liquid nitrogen overnight and stored at − 80 °C afterwards. None of the patients received pre-operative chemotherapy or radiation therapy. This study was approved and conducted in accordance with the policies of the Ethics Committee of the First Affiliated Hospital of Anhui Medical University. We obtained all patients’ informed consent.

### Cell culture

Human GC cell lines, such as AGS, MKN-28, SNU-1, HGC-27 and N87 were derived from the American Type Culture Collection (Manassas, VA, USA), and SGC-7901 and MGC-803 were purchased from Shanghai Cancer Institute (Shanghai, China). We purchased the gastric epithelial cell line GES-1 from the Cell Bank of Beijing Institute for Cancer Research (Beijing, China). RPMI-1640 medium supplemented with 10% fetal bovine serum was adopted for all cells culture. In addition, all cells were cultured at 37 °C in a humidified cell incubator with an atmosphere of 5% CO_2_.

### Quantification real-time PCR (qRT-PCR)

Total RNA was extracted from tumor and non-tumor samples human tissues or cultured cell liness by general protocol using Trizol kit (Invitrogen) TRIzol reagent (Invitrogen) based on the provided protocols. Total Extracted RNA (2 µg) was applied applicable for cDNA synthesis. Program settings are following: 25 °C 30 min, 42 °C 30 min, 85 °C 5 min, 4 °C 5 min. qRT-PCR analysis was performed using Hairpin-it TM microRNA and U6 snRNA Normalization RT-PCR Quantitation Kit (GenePharma Shanghai) based on vendor instructions. U6 was applied as an endogenous control for normalization. The following forward and reverse primers were used: miR-100-3p: Forward, 5′-TCGTTCGCTCAAGCTTGTATCTA-3′. Reverse, 5′-TATGGTTGTTCACCTCTCGTTCAC-3′, U6: Forward, 5′-CAGCACATATACTAAAATTGGAAGG-3′, Reverse, 5′-ACGAATTTGCGTGTCATCC-3′. For BMPR2 quantification, Probe qPCR Mix (RR391S, TaKaRa, USA) was used to monitor target gene amplification. All genes expression was normalized to GADPH. The following forward (F) and reverse (R) primers were used: BMPR2-F, 5′-CACCTCCTGACACAACACCACTC-3′, BMPR2-R, 5′-TGCTGCTGCCTCCATCATGTTC-3′; GAPDH-F, 5′-CCTTCATTGACCTCAACTAC-3′; GAPDH-R, 5′-CTCCTGGAAGATGGTGATGG-3′. All samples were analyzed in triplicates.

### Cell transfection

Lentiviral vectors used in this study (hsa-miR-100-3p mimic, hsa-miR-100-3p inhibitor and their control vectors) were purchased from Gene Pharma Company (Shanghai, China). AGS cells were infected by Lv-hsa-miR-100-3p-mimic or Lv-hsa-miR-100-3p-NC, MGC-803 cells were infected by Lv-hsa-miR-100-3p-inbibitor or Lv-hsa-miR-100-3p-NC. In brief, polybrene (6 μg/ml, Gene Pharma), along with the lentivrial vectors were supplemented into the cells at multiplicity of infection of 100–150 for 48 h. Then, survived cells were selected by puromycin (1–2 µg/ml) for 14 days. After that, healthy cell colonies were handpicked, and re-cultured in fresh cell culture medium and passaged for 3–5 times. The efficacy of lentiviral transduction was examined by qPCR on miR-100-3p expression levels in transduced cells. BMPR2 shRNA recombinant plasmid expression vector and over-expression recombinant plasmid vector pEX-3 (pGCMV/MCS/Neo) Homo BMPR2 (3117 bp) were all constructed by the Gene Pharma Company (Shanghai, China). The sequences of BMPR2 shRNA were: pGPU6-Neo-BMPR2-homo, 5′-GCTTGTGATGGAGTACTATCC-3′, pGPU6-Neo-shNC, 5′- GTTCTCCGAACGTGTCACGT-3′. The efficacy of infection was examined by Western blot.

### MTT assay

MTT Assay kit (KeyGentech, China) was used to measure cells viability according to the manufacturer’s protocol. In brief, when the cells were 80% confluence, the culture medium was aspirated and 20% MTT (200 μl) were added for 4 h incubation at 37 °C and the optical density value was then measured at 490 nm with a microplate reader (Thermo, USA).

### Annexin V-PE analysis

Cell apoptosis was analyzed in AGS and MGC-803 cells with miR-100-3p dysregulation by flow cytometry using the Annexin V-PE Apoptosis Detection Kit (BD Biosciences) in the presence or absence of cisplatin (2 µg/ml) for 48 h. Briefly, after different treatment by lentiviral vectors or cisplatin, the cells were dissociated and harvested. After 2 times washing with PBS, 500 µl flow buffer was used to cells resuspension. Finally, 5 µl Annexin V-FITC and 10 µl PI were respectively suppelemented for 15 min incubation. Flow cytometry was applied to apoptosis analysis.

### Plate cloning experiments

A total of 350 cells were cultured in the RPMI-1640 supplemented with 10% FBS supplementation. After 14 days culture at 37 °C in an atmosphere of 5% CO_2_, cells were then fixed with 4% paraformaldehyde for 30 min. Finally, crystal violet solution was responsible for nuclear staining after three times washing with phosphate-buffered saline (PBS).

### Migration and invasion assays

Transwell Boyden Chamber (Corning, Cambridge, MA, USA) was used for examination of cells movement. Upper chamber with or without matrigel coating (BD Biosciences) was used for migration and invasion assay respectively. Cells were plated into the upper compartment. After 12 h, cells accumulated in the lower chamber were washed, stained and imaged using microscopy (Olympus, Tokyo, Japan).

### Transcriptomic analysis and Bioinformatics analysis

We used the Illumina Xten platform to analyze gene expression differences. Differential gene GO of AGS cells with or without transfection by miR-100-3p mimic was analyzed using Illumina Xten platform (Sangon Biotech Shanghai Co., Ltd.). Targetscan prediction software was responsible for the screening of potential downstream target genes of miR-100-3p.

### Western blot

Cells were lysed by RIPA buffer (Beyotime, Shanghai, China). After determination of protein concerntration, each sample was loaded on SDS-PAGE for 50 min at 150 V and then was electro-transferred to a PVDF membrane (Millipore, Boston, MA, USA). After three times washing (TBST), target proteins were blocked with 5% bovine serum albumin for 1 h at room temperature. The membrane was then incubated with a primary antibody overnight at 4 °C. After three TBST washes, the membrane was incubated with corresponding horseradish peroxidase (HRP)-conjugated secondary antibody (Proteintech, USA) for 1 h at room temperature. Enhanced chemiluminescence kit was used to detect the signals with secondary antibody. The following antibodies were purchased from Cell Signaling Technology, Proteintech or AbcamThe antibodies used in this study were following: BMPR2 (CST, 6979); BMPR2 (R&D, AF811-SP); p-smad1/5/9 (CST, 13820); smad1/5/9 (Abcam, ab66737); caspase 3 (CST, 9662); bax (CST, 5023); bcl-2 (CST, 15071); p-Erk1/2 (CST, 4377); Erk1/2 (CST catalogue no., 4696); p-AKT (CST, 5012); AKT (CST, 2920); β-actin (Proteintech, 66009-1-Ig); anti-goat HRP-DAB Cell & Tissue Staining Kit (Brown, CTS008).

### Plasmid construction and genes interaction

Genomic DNA of GC cells was responsible for amplification of the 3′-UTR of BMPR2. Then the 3′-UTR of human BMPR2 was cloned into the XhoI/NotI sites of psi-CHECK2 (Promega, Madison, WI, USA) to generate the 3′-UTR wild-type reporter plasmid. Mutated 3′-UTR of BMPR2 was also cloned into psi-CHECK2 vector (250 pg/μl). Finally, these vectors were transfected in AGS cells (1 × 10^5^ cells/well). After 48 h transfection, luciferase signals were dectected and analysed by Luciferase assays kits (Promega) on a glomax-20/20 Luminometer (Promega).

### Immunohistochemistry staining

Paraffin section of gastric cancer tissues were applied to target proteins staining. Briefly, sections were deparaffinized through xylene and graded alcohol. Then, the citrate buffer was charged for antigen exposure. Subsequently, sections were incubated with primary antibodies, such as BMPR2 (R&D, AF811-SP) and Ki-67 (Abcam, ab15580). The next day, secondary antibodies labeled by HRP was used for target proteins detection.

### Tumor xenograft model

Nude mice with BALB/c background (male, 4–6 weeks), were purchased from Nanjing Model Animal center. These mice were used for tumorigenic assay. In brief, GC cells (5 × 10^6^ cells) with miR-100 inhibition or overexpression were established to model GC progression through subcutaneously injection. We measured the tumor size and mice weight twice a week. After 30 days, mice were sacrificed and tumor was harvested.

### Statistical analysis

Statistical analysis of all the data in this study were performed by prism 7 software (GraphPad Software, USA). Data was presented as mean ± SD from three independent experiments. Student’s t-test was used to analyze the significance between two groups. P values less than 0.05 were considered to be statistically significant.

## Results

### miR-100-3p is lowly expressed in GC cells

**Fig. 1:**
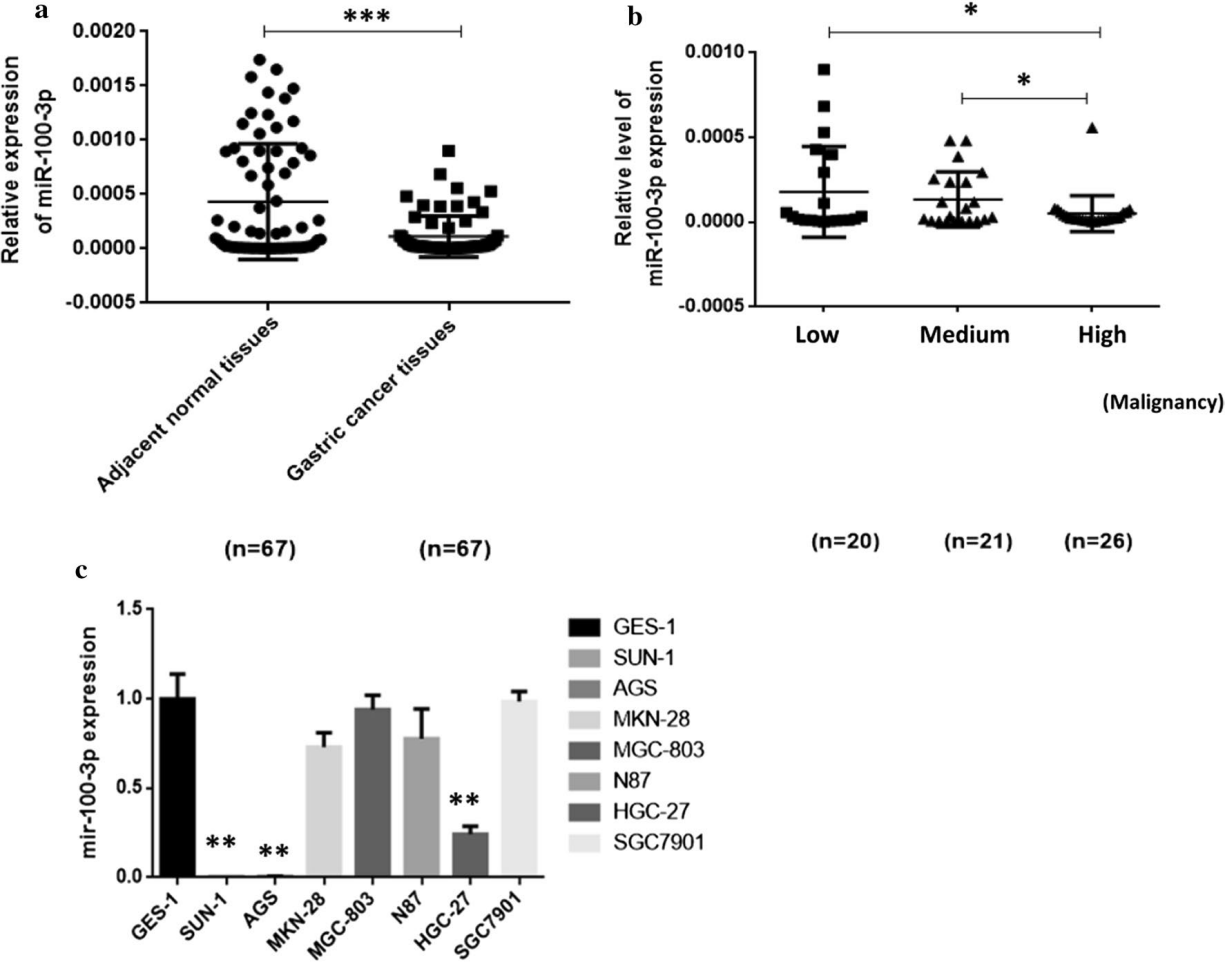
miR-100-3p was down-regulated in GC cells; **a** miR-100-3p was reduced in gastric cancer tissue.67 cases of fresh GC and corresponding normal tissues. **b** The miR-100-3p expression level is negatively correlated with the malignancy of GC. **c** The expression of miR-100-3p was dysregulated in GC cell lines. U6 small nuclear RNA gene (U6snRNA) was used as an internal control (* p < 0.05, *** p < 0.001)

To test the effect of miR-100-3p on GC, we first examined the expression of miR-100-3p in GC cells. qRT-PCR was used to measure the expression of miR-100-3p in 67 cases of fresh GC and corresponding normal tissues. We found that miR-100-3p was significantly down-regulated in GC compared to in normal adjacent tissues (Fig. 1a). In addition, miR-100-3p expression level was positively correlated with the malignant degree of GC (Fig. 1b and Table 1). We also screened the expression of miR-100-3p in various GC cell lines. Compared with the immortalized gastric mucosal epithelial cells GES-1, miR-100-3p was significantly down-regulated in AGS, SUN-1 and HGC-27 cells, but MGC803, MKN-28, N87 and SGC-7901 cells did not showed altered miR-100-3p expression (Fig. 1c). These results supported the idea that miR-100-3p might be a tumor suppressor in GC.

**Table 1:** Clinicopathologic characteristics of gastric cancer associated with miR-100-3p

Variables	N	t or F value	P value
Age (year)		1.954	0.0540
≥ 60	53		
< 6	14		
Gender		1.089	0.2800
Male	49		
Female	18		
Tumor size (cm)		1.623	0.1095
≥ 5 cm	32		
< 5 cm	35		
Histological classification		2.88	0.0303
Well-Moderately differentiated	20		
Moderately–Poorly differentiate	21		
Poorly differentiate	26		
Lymph-node metastasis		0.4838	0.6301
Yes	43		
No	24		
TNM Stage		0.3633	0.7176
I+II	24		
III+IV	43		
Vascular invasion		1.338	0.1855
Yes	26		
No	41		

### miR-100-3p inhibited GC cells growth, migration, invasion and promoted apoptosis

**Fig. 2:**
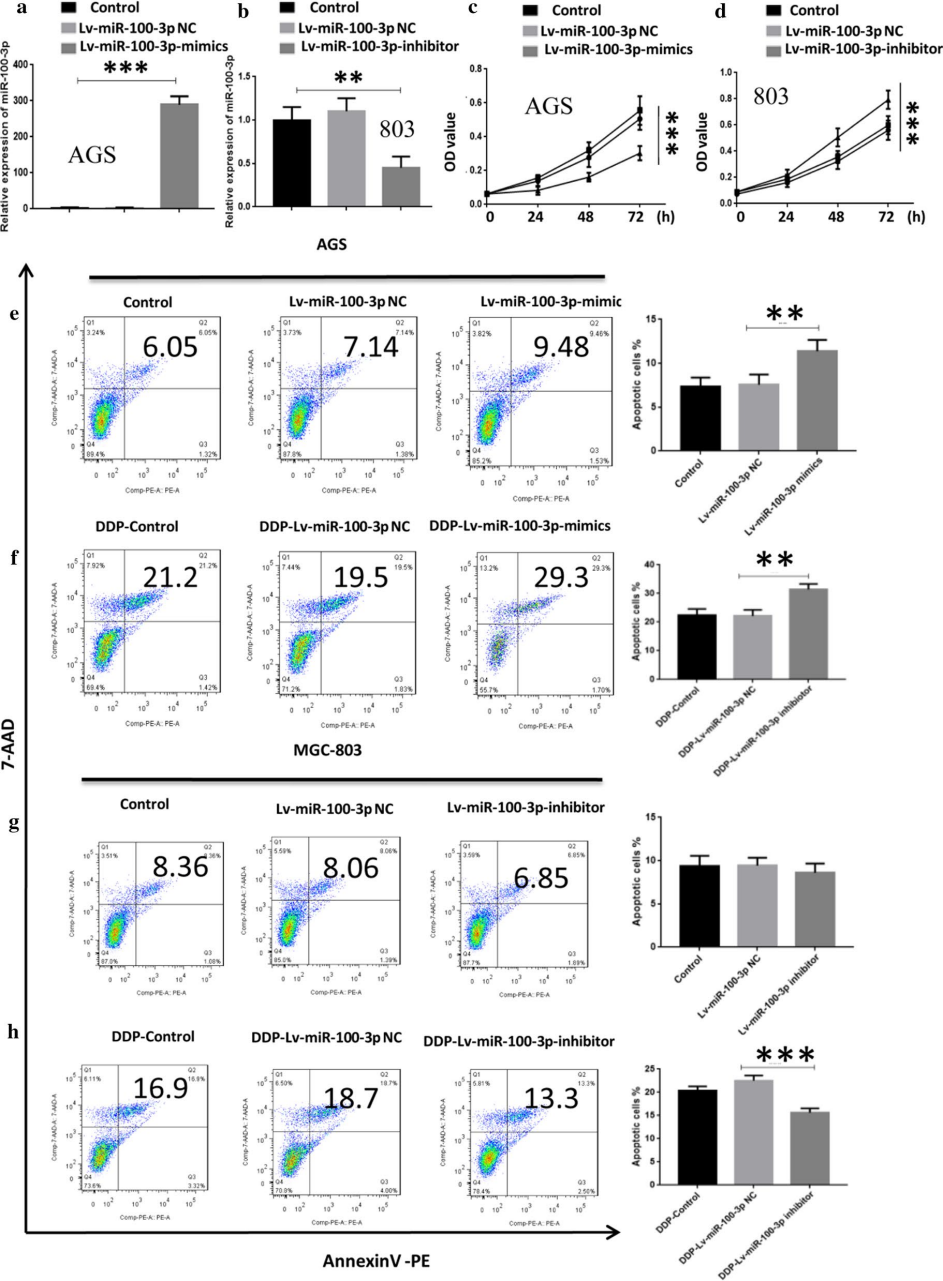
Over-expression of miR-100-3p alleviated the malignancy of AGS cells, but suppression of miR-100-3p promoted malignancy of GC cells. **a**, **b** qRT-PCR results showed the efficiency of transfection. **c**, **d** MTT assay indicated the proliferation of AGS and MGC-803 cells with miR-100-3p inhibition or overexpression. **e**–**h** Apoptotic assay showed the apoptosis of GC cell lines, in the absence or presence of DDP (2 μg/ml) (*p < 0.05, **p < 0.01, ***p < 0.001, ****p < 0.0001)

To further analyze the function of miR-100-3p in GC, we established the GC cells with miR-100-3p overexpression or inhibition. MGC-803 cells, characterized by higher expression of miR-100-3p, were used for miR-100-3p knockdown to check the effect of miR-100-3p inhibition on GC cells biology. AGS cells, possessing lower expression of miR-100-3p, were infected with miR-100-3p mimic to examine whether revovery of miR-100 affected GC cells biology. Efficiency of transfection was measured using qRT-PCR (Fig. 2a). Results showed that cell growth was suppressed in AGS cells with the miR-100-3p overexpression (Fig. 2c). In addition, we found that the colony formation and invasion ability of AGS cells transfected with Lv-miR-100-3p mimic was lower those that of the untransfected and negative control groups (Additional file 1: Figs. S1A and S2A). Instead, the colony forming and invasion ability of MGC803 cells was higher than those of the untransfected and the negative control groups (Additional file 1: Figs. S1A and S2B). Annexin V-PE staining results showed that miR-100-3p overexpression induced cell apoptosis at 48 h in AGS cells (Fig. 2e). We then tested whether miR-100-3p inhibition affected GC proliferation. Results showed the cell growth was promoted in MGC-803 cells with miR-100-3p inhibition (Fig. 2d). Moreover, miR-100-3p inhibition suppressed cell apoptosis at 48 h in MGC-803 cells (Fig. 2g). miR-100-3p inhibition could also weaken the pro-apoptotic effects of Cisplatin (DDP 2 µg/ml), a common chemotherapy drug to treat GC. The cells were treated with or without DDP for 48 h, and we observed that miR-100-3p suppression inhibited apoptosis induced by DDP treatment (Fig. 2f, h).

### MiR-100-3p directly targets to BMPR2

**Fig. 3:**
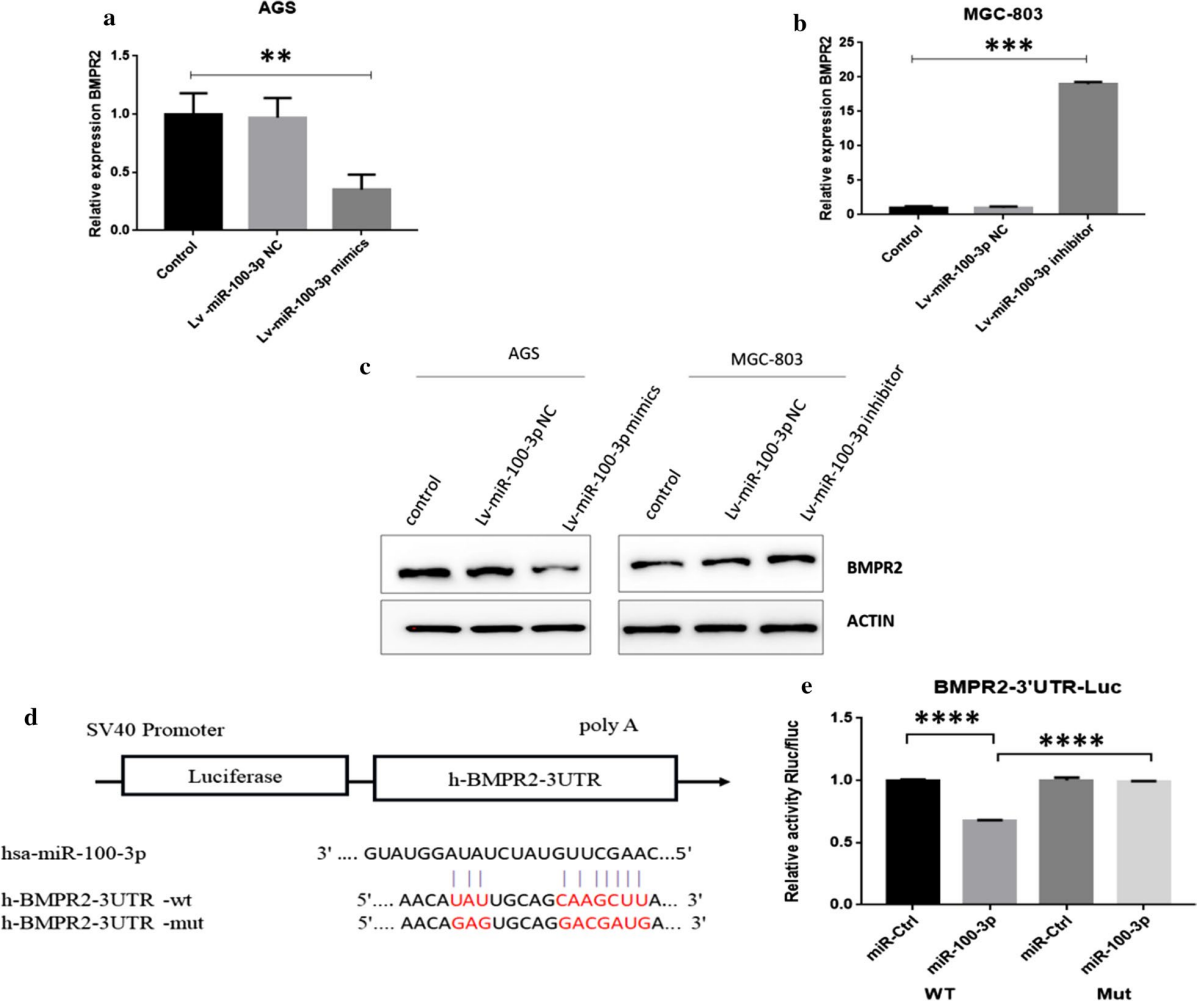
miR-100-3p targets to the BMPR2. **a**, **b** qRT-PCR showed the expression of BMPR2 after transfection of miR-100-3p mimic or inhibitor. **c** Western blot indicated the protein expression of BMPR2 after miR-100-3p overexpression or inhibition. (D) Conserved binding site of BMPR2 and miR-100-3p. **e** Luciferase activity assay confirmed the interaction between BMPR2 and miR-100-3p (**p < 0.01, ****p < 0.001)

To examine the underlying mechanism of underlying dysregulation of miR-100-3p in GC, we used transcriptomics sequencing to detect changes in transcripts before and after transfection of miR-100-3p mimic in AGS cells. The results showed that there were 222 down-regulated genes and 36 up-regulated genes (Additional file 1: Fig. S3A), BMPR2 was one of the most prominently down-regulated genes (ranked fourth) (Additional file 1: Fig. S3A, B). At the same time, BMPR2 was significantly predicted poor survival of GC patients. We further analyzed the correlation between BMPR2 and miR-100-3p expression in 42 GC patients, and found a significant negative correlation between them (Additional file 1: Fig. S3C). Thus, we hypothesized miR-100-3p could directly regulate BMPR2 expression. We next examined the effect of whether miR-100-3p affected BMPR2 expression. miR-100-3p mimic was transfected into AGS cells and we found BMPR2 was dramatically down-regulated (Fig. 3a). Conversely, miR-100-3p inhibition promoted BMPR2 expression in MGC-803 cells (Fig. 3b). Consistently, miR-100-3p overexpression reduced BMPR2 protein level and the opposite was also true (Fig. 3c). Evidence so far suggested BMPR2 could be a downstream target of miR-100-3p. Therefore, we checked the interaction of miR-100-3p and BMPR2. Results showed, after transfection of miR-100-3p, the cells transfected by WT BMPR2 showed higher luciferase activity, but mutant BMPR2-expressing cells showed no luciferase activity (Fig. 3d, e). Overall, these data provided evidence that BMPR2 was a direct target of miR-100-3p and was negatively regulated by miR-100-3p.

### BMPR2 mRNA was highly expressed in fresh GC samples and negatively correlated with patient survival

**Fig. 4:**
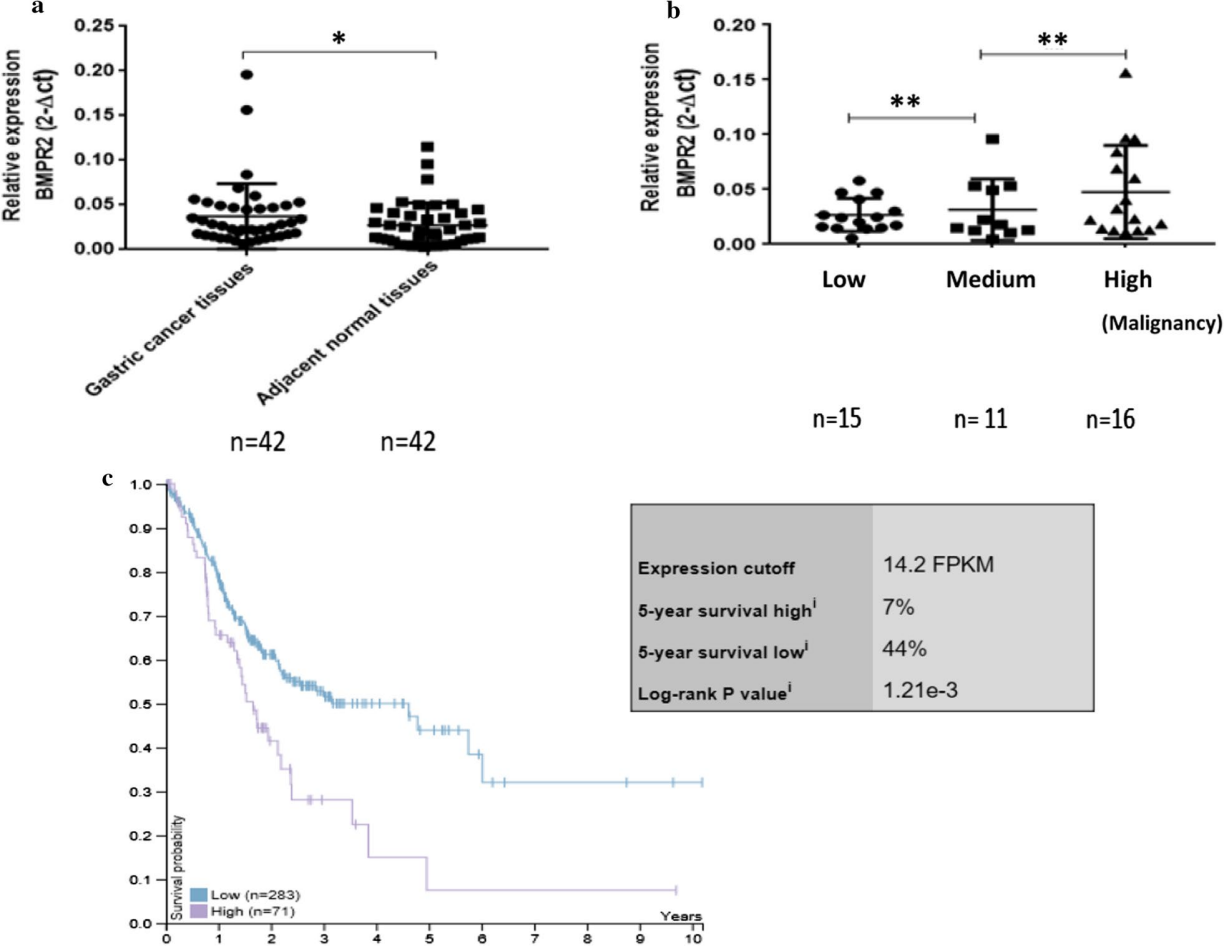
BMPR2 was up-regulated in most of the fresh specimens from GC patients; higher expression BMPR2 has lower survival. **a** 42 cases of fresh GC and corresponding normal tissues; **b** the BMPR2 mRNA expression level is correlation with the pathological type of GC, the worse the differentiation, the high the expression level. GADPH was used as an internal control. **c** Higher expression BMPR2 has lower survival (*p < 0.05 ** p < 0.01)

To confirm the function of BMPR2 in GC, we next analyzed the expression of BMPR2 in GC. qRT-PCR results showed the expression levels of BMPR2 in 42 cases of fresh GC and corresponding normal tissues (Fig. 4a). In addition, BMPR2 expression level was positively correlated with the malignant degree of GC (Fig. 4b). We also used TCGA database to analyze the relationship between BMPR2 and GC survival (Table 2), and found that GC patients with high expression of BMPR2 showed shorter survival than those with low expression (Fig. 4c).

**Table 2:** Gastric cancer patient information from the TCGA database

Total number of gastric cancer cases	354
Survival state	
Survive	208
Death	146
Gender	
Male	229
Female	125
Clinical stage	
I	2
Ia	13
Ib	33
II	27
IIa	34
IIb	49
III	3
IIIa	59
IIIb	51
IIIc	33
IV	35
n/a	15

### BMPR2 reversed the malignancy caused by miR-100-3p inhibition

**Fig. 5:**
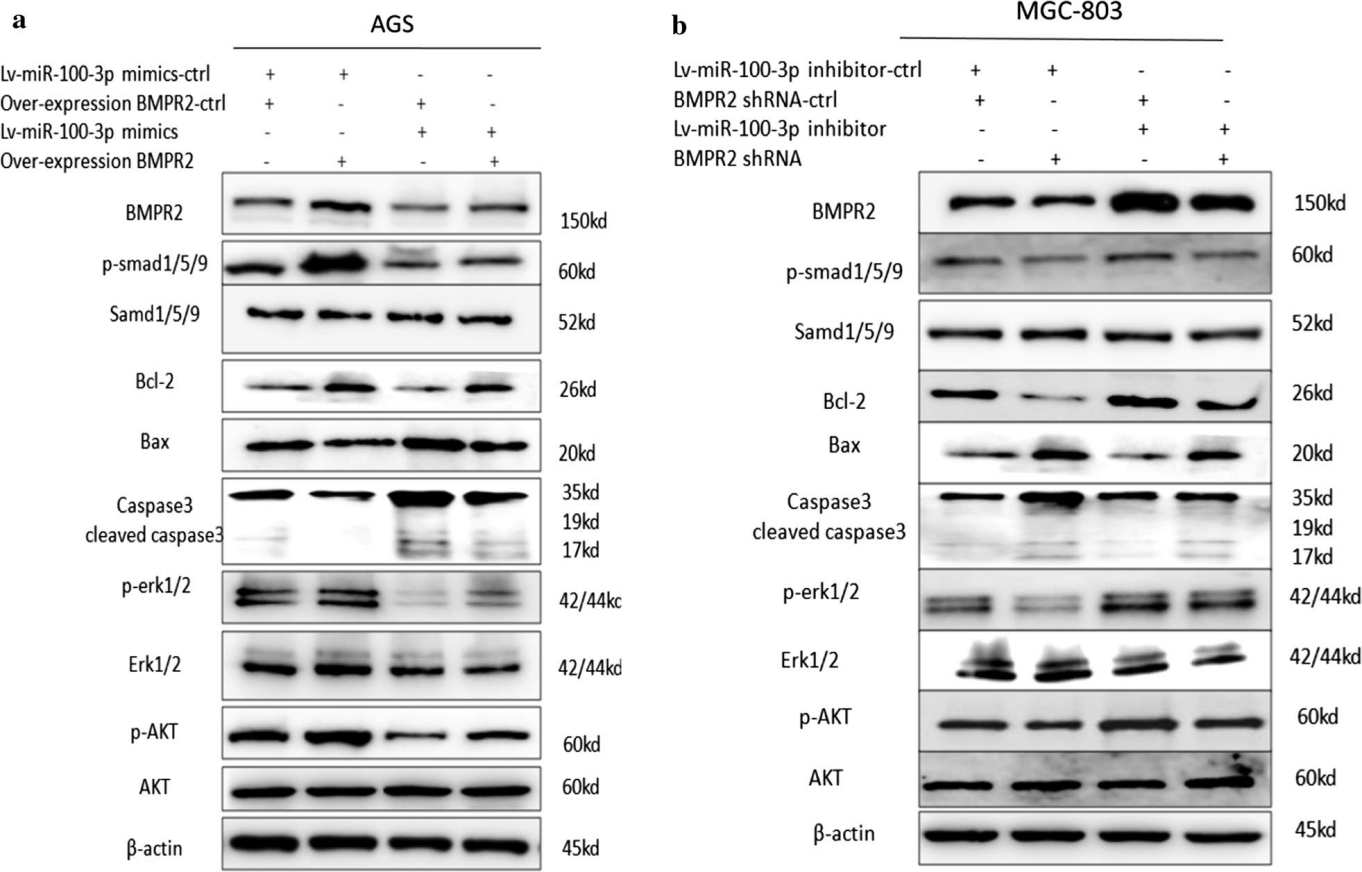
miR-100-3p functioned through BMPR2 in inhibiting Smad1/5/9、ERK-AKT and Bcl-2 signaling. **a** Western blotting for Smad1/5/9, p-smad1/5/9, Bcl-2, Bax, Caspase3, ERK1/2, p-EKR1/2, AKT and p-AK in AGS cells transfected with Lv-miR-100-3p mimic-control (Lv-miR-100-3p mimic-ctrl) and over-expression BMPR2 plasmid control(over-expression BMPR2 control-ctrl), Lv-miR-100-3p mimic-ctrl and over-expression BMPR2 plasmid, Lv-miR-100-3p mimic and over-expression BMPR2-ctrl, or Lv-miR-100-3p mimic and over-expression BMPR2 plasmid. **b** MGC-803 cells transfected with Lv-miR-100-3p inhibitor-control (inhibitor-ctrl) and BMPR2 shRNA control (shRNA-ctrl), Lv-miR-100-3p inhibitor-ctrl and BMPR2 shRNA, Lv-miR-100-3p inhibitor and BMPR2 shRNA-ctrl, or Lv-miR-100-3p inhibitor and BMPR2 shRNA. Protein expression was quantified by band intensity and normalized to β-actin. **p < 0.01

To further confirm the interaction of BMPR2 and miR-100-3p in GC, BMPR2 was overexpressed in GC cells with miR-100-3p inhibition or inhibited in GC cells expressing miR-100-3p mimic. As expected, miR100-3p inhibition increased BMPR2 expression, while miR-100-3p mimic showed the opposite effect. Notably, these effects were rescued by exogenous BMPR2 interference (Fig. 5a, b). As BMPR2 was reported to activate the Smad1/5/9, ERK-AKT and Bcl-2 signaling pathways [13–18], we tested whether miR-100-3p could also regulate these pathways by targeting BMPR2. We found that inhibition of miR-100-3p loss significantly increased promoted phosphorylation of phosphor-Smad1/5/9, phosphor-ERK1/2, and phosphor-AKT and as well as Bcl-2 levels expression, while decreased expression of Bax, Caspase3 and cleaved caspase3 was observed in MGC-803 cells. Vice versa, over-expression of miR-100-3p significantly decreased phosphor-smad1/5/9, phosphor-ERK1/2, phosphor-AKT and Bcl-2 levels, while increased Bax, Caspase3 and cleaved caspase3 in AGS cells. Moreover, BMPR2 siRNA (siBNMPR2) significantly restored Smad1/5/9, ERK-AKT and Bcl-2 activity in miR-100-3p inhibited MGC-803 cells, while BMPR2 over-expression significantly restored Smad1/5/9, ERK-AKT and Bcl-2 activity in miR-100-3p over-expressing AGS cells (Fig. 5a, b). These data further supported the notion that BMPR2 was a downstream functional mediator of miR-100-3p.

**Fig. 6:**
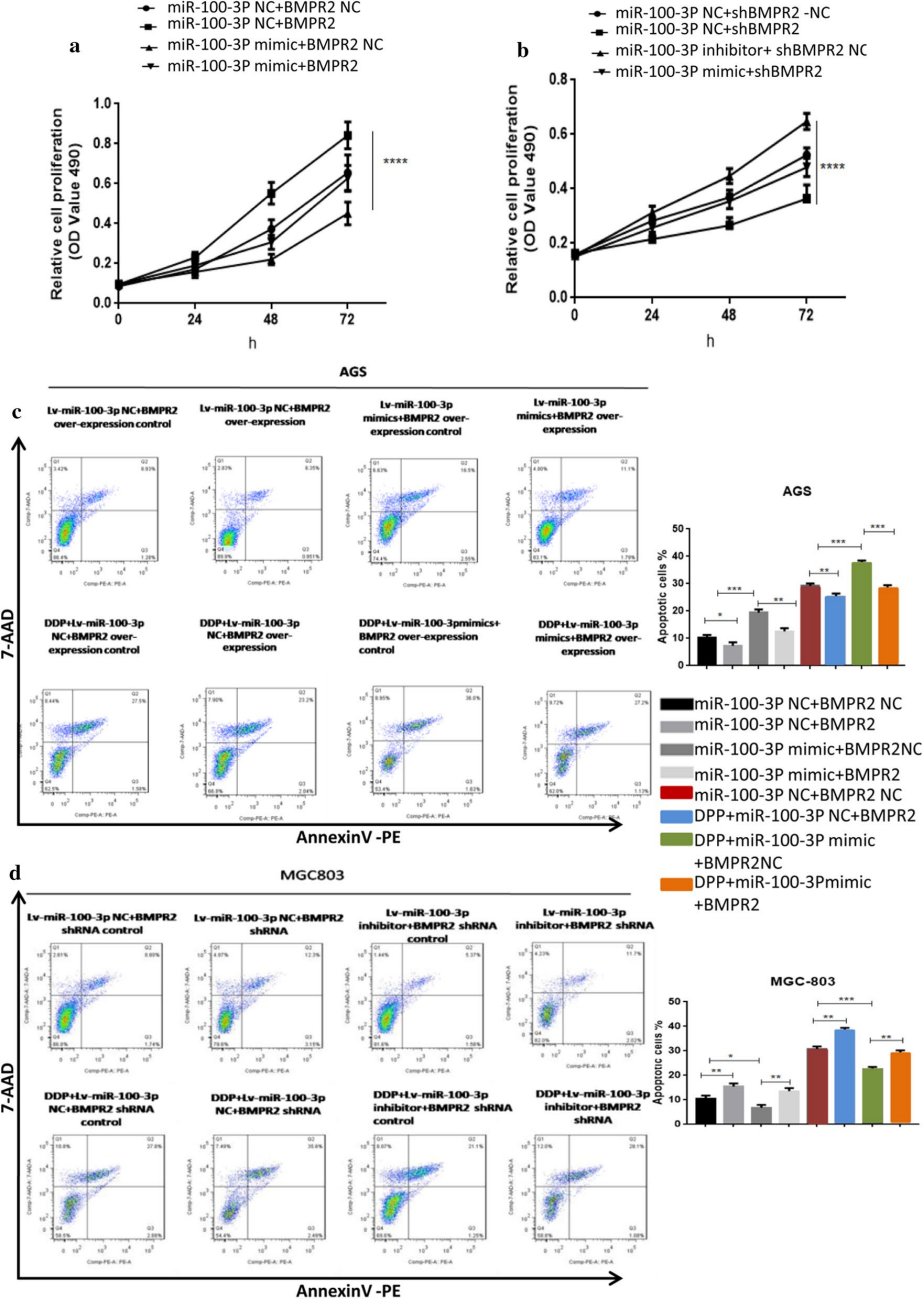
Knockdown of BMPR2 expression neutralized the effect of the miR-100-3p inhibitor; also over-expression of BMPR2 neutralized the effect of the miR-100-3p over-expression. BMPR2 reversed the effect induced by miR-100-3p dysregulation. **a**, **b** Proliferation of AGS cells transfected with Lv-miR-100-3p mimic-control (Lv-miR-100-3p mimic-ctrl) and over-expression BMPR2 plasmid control (over-expression BMPR2 control-ctrl), Lv-miR-100-3p mimic-ctrl and over-expression BMPR2 plasmid, Lv-miR-100-3p mimic and over-expression BMPR2-ctrl, or Lv-miR-100-3p mimic and over-expression BMPR2 plasmid. MGC-803 cells transfected with Lv-miR-100-3p inhibitor-control (inhibitor-ctrl) and BMPR2 shRNA control (shRNA-ctrl), Lv-miR-100-3p inhibitor-ctrl and BMPR2 shRNA, Lv-miR-100-3p inhibitor and BMPR2 shRNA-ctrl, or Lv-miR-100-3p inhibitor and BMPR2 shRNA was examined by MTT assay **c**, **d** Flow analysis revealed the apoptosis of AGS and MGC-803 cells with or without DDP supplementation (*p < 0.05, **p < 0.01, ***p < 0.001, ****p < 0.0001)

We next investigated whether miR-100-3p could repress BMPR2 to regulate GC cells function. Expectedly, Knockdown of miR-100-3p significantly increased the proliferation of MGC-803 cells, while over-expression of miR-100-3p suppress the proliferation of AGS cells (Fig. 6a, b). Moreover, transfection of Si-BMPR2 or BMPR2 reversed this effect (Fig. 6a, b). Consistently, this phenomenon was applied to GC cells apoptosis (Fig. 6c, d). Additionally, following DDP treatment, siBMPR2 attenuated miR-100-3p suppression inhibited apoptosis in MGC-803 cells, while over-expression of BMPR2 attenuated miR-100-3p over-expression induced apoptosis in AGS cells. Overall, these results indicate miR-100-3p/BMPR2 signaling is critical to GC cells malignant biology.

### miR-100-3p inhibited tumor growth through BMPR2 in vivo

**Fig. 7:**
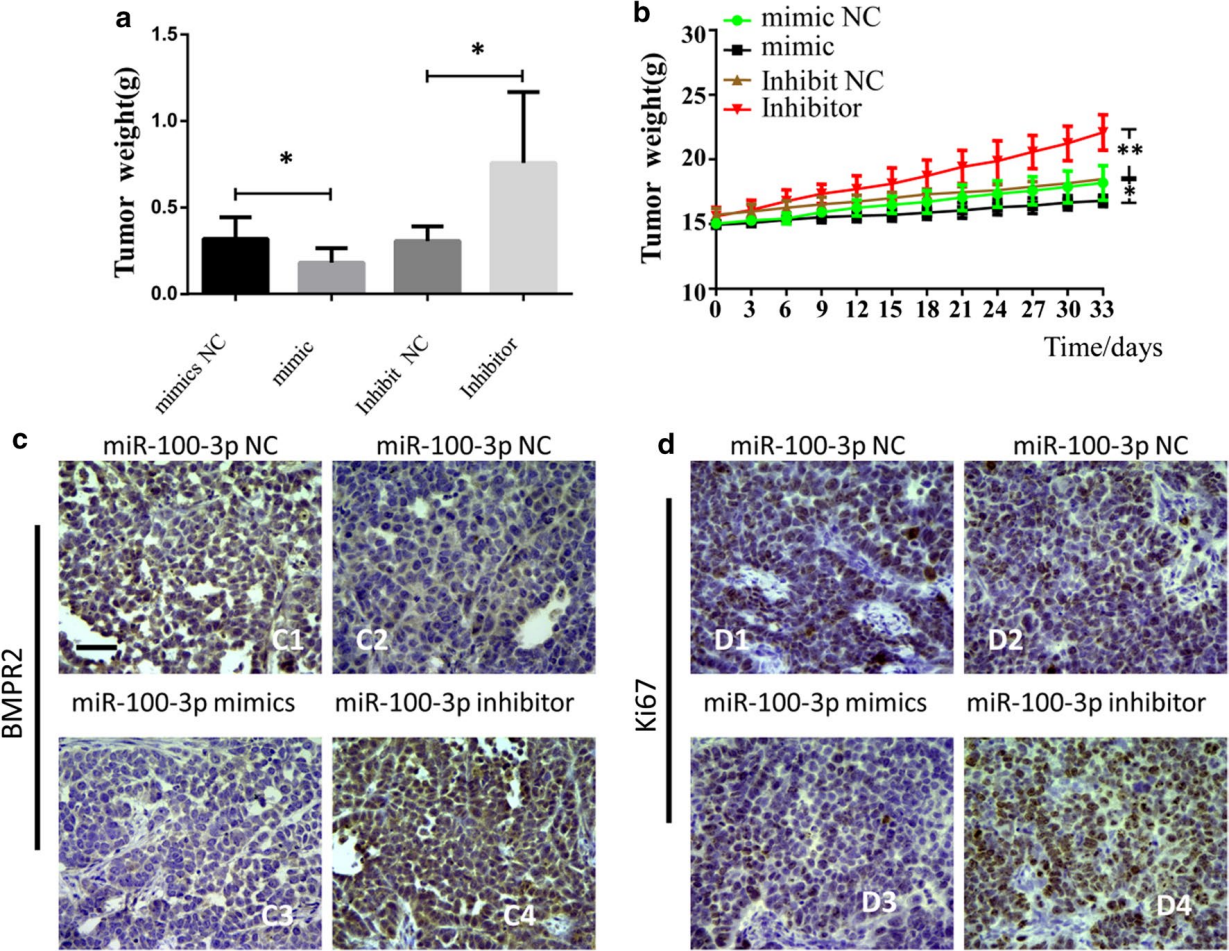
miR-100-3p inhibited tumor growth through BMPR2 in vivo. **a**, **b** represented figure indicated the tumor growth after miR-100 overexpresison or inhibition. **c**, **d** immune staining indicated the expression of BMPR2 and Ki-67, scale bar = 200 μm

To confirm the function of miR-100-3p in vivo, we established xenograft tumor model in nude mice through subcutaneously injection of GC cells with miR-100-3p over-expression or inhibition. Results showed miR-100-3p suppressed, while inhibition of miR-100-3p promoted tumor growth (Fig. 7a, b). Moreover, in tumor cells with miR-100-3p inhibition, more ki67 and BMPR2 positive cells were observed (Fig. 7c, d).

## Discussion

Herein, we found that miR-100-3p expression was lower in both GC patient samples and cell lines compare to controls. Interestingly, our results indicated that miR-100-3p was curical for GC cells proliferation, migration and invasion. Furthermore, we found that miR-100-3p mimic increased the sensitivity of AGS cells to the chemotherapeutic agent DDP, and miR-100-3p inhibitor decreased the sensitivity of MGC-803 cells to the chemotherapeutic agent DDP. Overall, these data indicated that miR-100-3p is a tumor suppressor in GC.

Furthermore, our results indicated that BMPR2 functions as a key downstream target gene of miR-100-3p. BMPR2, a member of the BMP receptor family of transmembrane serine/threonine kinases, is a key molecule in BMP signaling. Upon binding to BMP, BMPR2 is phosphorylated and activates BMPR1, which in turn leads to the phosphorylation of intracellular Smad1, 5 and 8. Subsequently, the common mediator Smad4 binds to phosphorylated Smad1, 5 and 8 and is translocated into the nucleus, where it activates the transcription of BMPR target genes [19, 20]. BMPR2 inactivating mutations cause pulmonary arterial hypertension [19, 21, 22]. Recently, BMPR2 was reported with contradicting functions in cancers. BMPR2 promoted human osteosarcoma cell invasion and metastasis through the RhoA-Rocklimk2 pathway [23]. In contrast, BMPR2 downregulation promoted the development of neuroblastoma [24]. In human chondrosarcoma cells, BMPR2 inhibited apoptosis and autophagy through destabilization of XIAP [25]. In acute myeloid leukemia, SPG6 supported development of acute myeloid leukemia by regulating BMPR2-Smad-Bcl-2/Bcl-xl signaling [18]. Previous studies have revealed that, in osteogenic differentiation of human mesenchymal stem cells, miR-23a suppresses the expression of BMPR2 in human pulmonary artery smooth muscle cells, and BMPR2 is also regulated by miR-1152 [26, 27]. Obviously, BMPR2 was regulated by a great deal of miRNAs in the context of cell types. However, the dynamic progress in miR-100 mediated BMPR2 regulation is still largely unclear.

In this study, herein, we found that BMPR2 was found that it could be a directly target of to miR-100-3p by luciferase reporter assay and qPCR, and Western blot with samples from GC cell lines further supported the idea. Over-expressionintroduction of miR-100-3p in GC cells inhibited proliferation and promoted apoptotic apoptosis of GC cells, which were attenuated by over-expression of BMPR2. Knockdown of miR-100-3p promoted proliferation and inhibited apoptotic of GC cells, which were attenuated by the siRNA mediated suppression of BMPR2. We used TCGA database to conduct a Kaplan–Meier survival analysis of GC patients, and found that, compared with patients with lower BMPR2 expression, patients with higher expression of BMPR2 had worse overall survival. These data suggested that the miR-100-3p/BMPR2 axis was critical to GC, and miR-100-3p was a tumor suppressor miRNA.

BMPR2 has been found to be implicated in diverse disease conditions, such as human osteosarcoma metastasis [23, 28] and pulmonary arterial hypertension [29], suggesting that dysregulation of BMPR2 might contribute to disease progression. Furthermore, the association between miR-100-3p and ERK/AKT and Bax-Bcl2-Caspase3 pathways was investigated, and the results showed that over-expression of miR-100-3p inhibited the ERK/AKT pathway and activated the Bax-Bcl2-Caspase3 pathway, whereas knockdown of miR-100-3p activated the ERK/AKT pathway and inhibited the Bax-Bcl2-Caspase3 pathway, likely via BMPR2.

miRNA dysregulation has been reported to be valuable for GC diagnosis [30–32]. Recently, several studies even demonstrated the potential of miRNA in GC diagnosis and treatment [33, 34]. Here, we proposed that up-regulation of miR-100-3p could inhibit proliferation and enhance apoptosis of GC cells. Therefore, combining therapies targeting miR-100-3p with existing conventional treatments may be novel strategy against GC.

## Conclusion

In summary, we identified, for the first time, that the tumor suppressor miRNA miR-100-3p directly targeted to BMPR2 and consequently regulated the ERK/AKT and Bax/Bcl2-Caspase3 signaling pathways in GC. These findings are valuable for both basic medical research and clinical applications.

## Supplementary information

**Additional file 1: Fig. S1.** miR-100 dysfunction controls gastric cancer cells proliferation. (A) Over-expression miR-100-3p in AGS cells inhibited AGS cells clone formation ability, scale bar = 200 μm (B) know-down miR-100-3p in MGC-803 cells promoted MGC803 cells clone formation ability, scale bar =200 μm ( *** p < 0.001). **Fig. S2.** Over-expression miR-100-3p in AGS cells suppressed the migration and invasion of AGS cells. Know-down miR-100-3p in MGC-803 cells promoted the migration and invasion of MGC803 cells. (A) Invasion assay. (B) Migration assay (*** p < 0.01, **** p < 0.01). **Fig. S3.** Transcriptome changes before and after transfection of miR-100-3p mimic in AGS cells. (A) Comparison group expression difference scatter plot, red indicates up-regulated genes, green indicates down-regulated genes, and black indicates non-differentiated genes. (B) Target DE down-regulated gene TPM heat map (Top50). (C) Correlation analysis of expression of miR-100-3p and BMPR2 in GC tissues (** p < 0.01).

## Data Availability

The datasets used and/or analyzed during the current study are available from the corresponding author on reasonable request.
