# Supplementary material for: miR-100-3p inhibits cell proliferation and induces apoptosis in human gastric cancer through targeting to BMPR2
Source: Cancer Cell Int. 2019 Dec 27;19:354. doi: 10.1186/s12935-019-1060-2 (PMC6935118; doi:10.1186/s12935-019-1060-2)
Supplement: Supplementary file 1 — Additional file 1: Fig. S1. miR-100 dysfunction controls gastric cancer cells proliferation. (A) Over-expression miR-100-3p in AGS cells inhibited AGS cells clone formation ability, scale bar = 200 μm (B) know-down miR-100-3p in MGC-803 cells promoted MGC803 cells clone formation ability, scale bar =200 μm ( *** p < 0.001). Fig. S2. Over-expression miR-100-3p in AGS cells suppressed the migration and invasion of AGS cells. Know-down miR-100-3p in MGC-803 cells promoted the migration and invasion of MGC803 cells. (A) Invasion assay. (B) Migration assay (*** p < 0.01, **** p < 0.01). Fig. S3. Transcriptome changes before and after transfection of miR-100-3p mimic in AGS cells. (A) Comparison group expression difference scatter plot, red indicates up-regulated genes, green indicates down-regulated genes, and black indicates non-differentiated genes. (B) Target DE down-regulated gene TPM heat map (Top50). (C) Correlation analysis of expression of miR-100-3p and BMPR2 in GC tissues (** p < 0.01). [file 12935_2019_1060_MOESM1_ESM.docx]

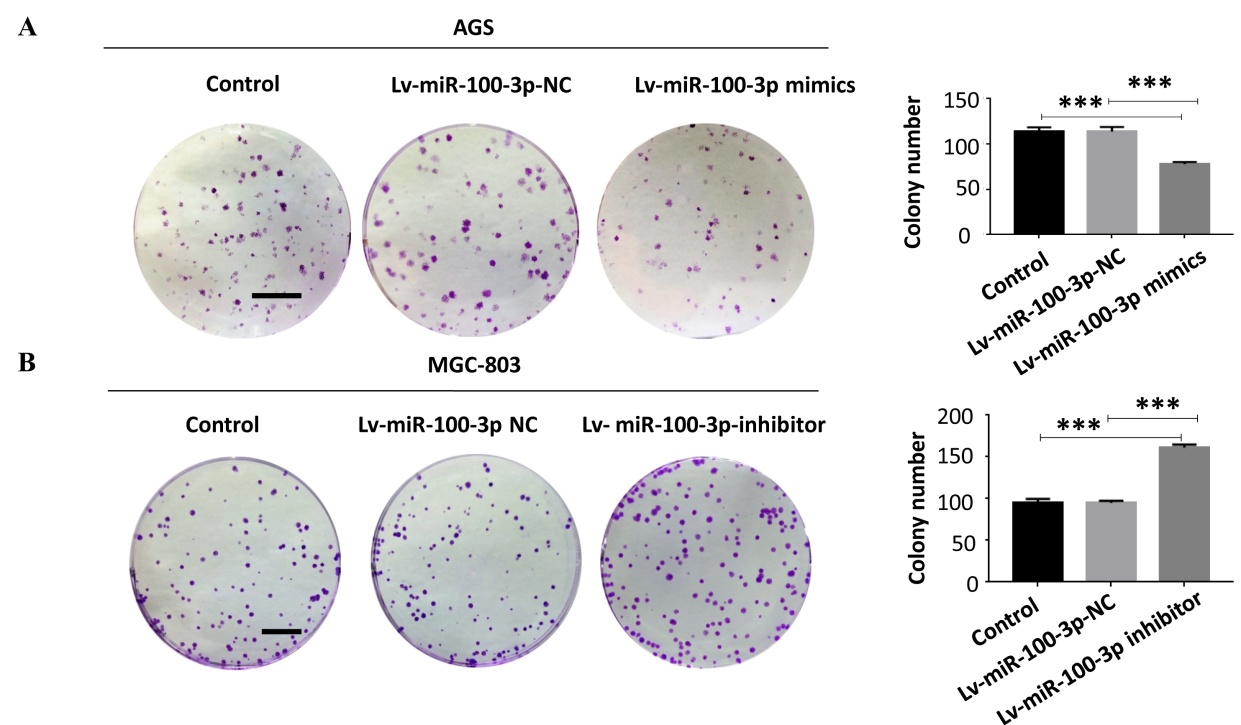


**Fig. S1. miR-100 dysfunction controls gastric cancer cells proliferation. (A)** Over-expression miR-100-3p in AGS cells inhibited AGS cells clone formation ability, scale bar = 200 μm (**B**) know-down miR-100-3p in MGC-803 cells promoted MGC803 cells clone formation ability, scale bar =200 μm. ( *** p<0.001)


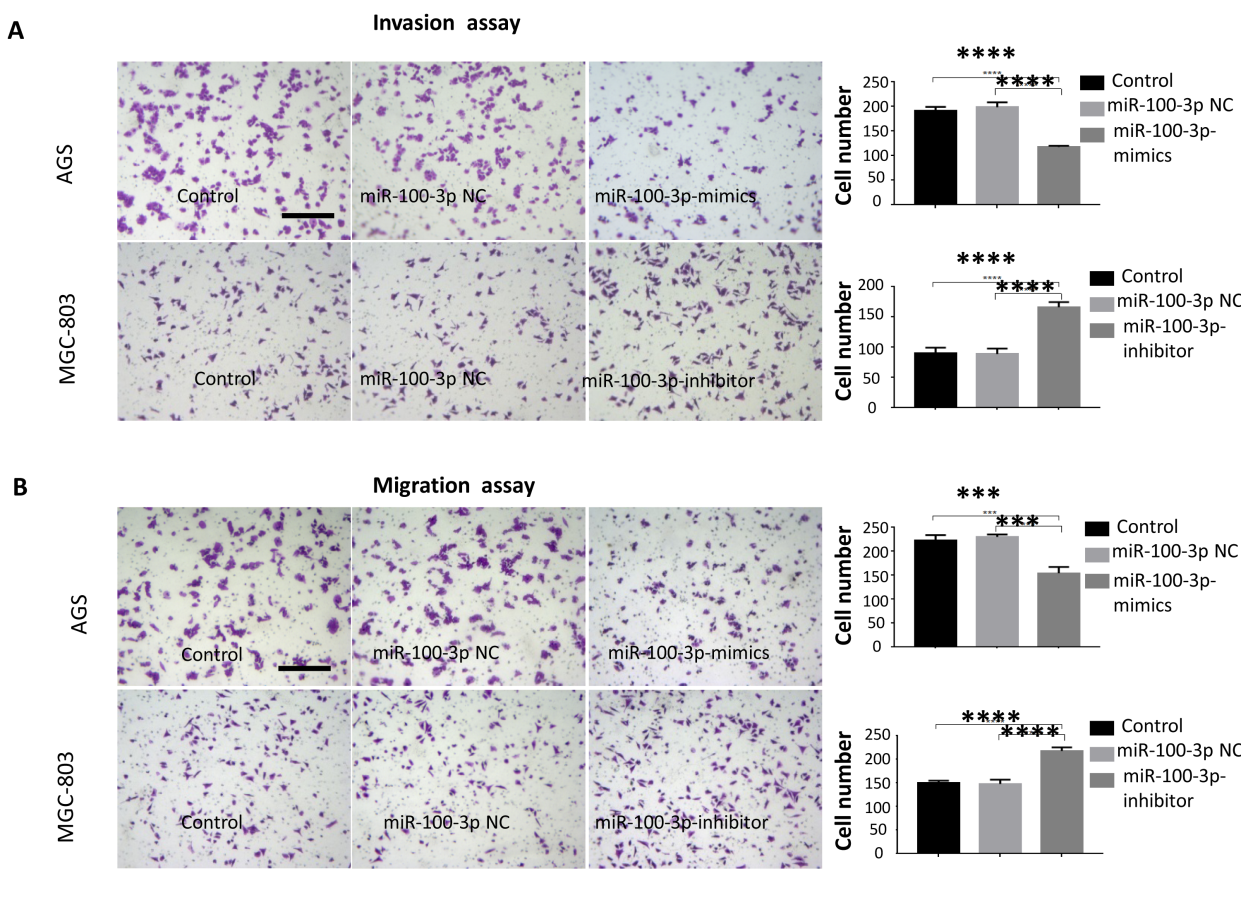


**Fig. S2 Over-expression miR-100-3p in AGS cells suppressed the migration and invasion of AGS cells.** Know-down miR-100-3p in MGC-803 cells promoted the migration and invasion of MGC803 cells. (**A)** Invasion assay. **(B)** Migration assay. (*** p <0.01,**** p <0.01)


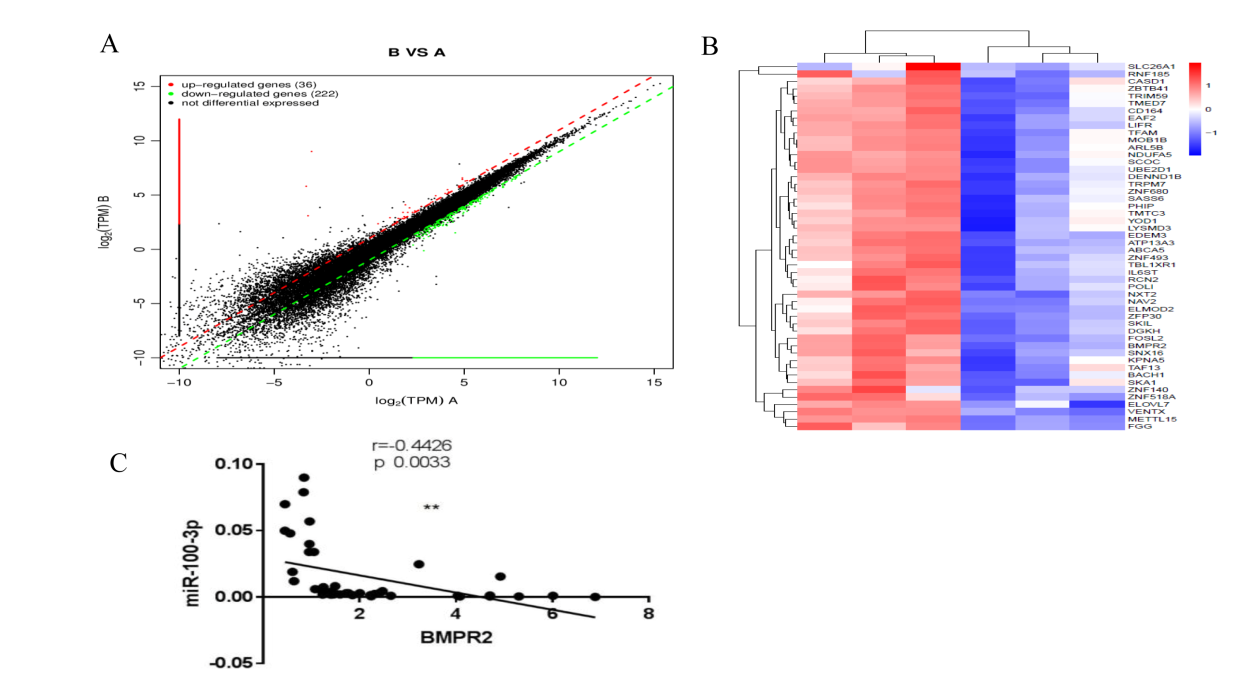


**Fig. S3 Transcriptome changes before and after transfection of miR-100-3p mimic in AGS cells.** (**A)** Comparison group expression difference scatter plot, red indicates up-regulated genes, green indicates down-regulated genes, and black indicates non-differentiated genes. (**B)** Target DE down-regulated gene TPM heat map (Top50). **(C)** Correlation analysis of expression of miR-100-3p and BMPR2 in GC tissues. (** p <0.01)
